# Impact of Refeeding Syndrome on the Short-Term Clinical Outcomes of Very-Premature Infants

**DOI:** 10.3390/nu16203445

**Published:** 2024-10-11

**Authors:** Mountasser M. Al-Mouqdad, Belal Alshaikh, Haider H. Sumaily, Ameen A. Almotiri, Nabeel A. Alodhaidan, Latifah AlMahmoud, Adli Abdelrahim, Tamadur E. Yousif, Abdullah S. Alghamdi, Yasir A. Albarrak, Aljohara O. Alnafiey, Maha R. Al-Anazi, Thanaa M. Khalil, Raneem S. Asfour, Suzan S. Asfour

**Affiliations:** 1 Neonatal Intensive Care, Hospital of Pediatrics, King Saud Medical City, Riyadh 12746, Saudi Arabia; 2 Department of Pediatrics, Cumming School of Medicine, University of Calgary, Calgary, AB T2N 1N4, Canada; 3 Pediatric Gastroenterology Department, King Saud Medical City, Riyadh 12746, Saudi Arabia; 4 Pharmacy Department, Pharmaceutical Care Services, King Saud Medical City, Riyadh 12746, Saudi Arabia; 5 Obstetric and Gynecology Department, Maternity Hospital, King Saud Medical City, Riyadh 12746, Saudi Arabia; 6 Pharmacy College, Jordan University of Science and Technology, P.O. Box 3030, Irbid 22110, Jordan; 7 Clinical Pharmacy Department, Pharmaceutical Care Services, King Saud Medical City, Riyadh 12746, Saudi Arabia

**Keywords:** parenteral nutrition, refeeding syndrome, mortality, intraventricular hemorrhage, preterm infants

## Abstract

Background: Refeeding syndrome (RFS) is a potentially life-threatening condition that can occur in preterm infants if nutritional support is initiated or increased after a period of starvation or malnutrition. Objectives: The current study aimed to examine the short-term clinical outcomes of RFS in preterm infants born at ≤32 weeks of gestation. Methods: Infants with a gestational age of ≤32 weeks and a birth weight of <1500 g who were born and admitted to the level III neonatal intensive care unit and received parenteral nutrition upon admission were retrospectively evaluated. The modified log Poisson regression with generalized linear models and a robust variance estimator was applied to adjust the outcomes of infants. Results: In total, 760 infants met this study’s inclusion criteria. Of them, 289 (38%) developed RFS. RFS was significantly associated with a composite outcome of mortality and intraventricular hemorrhage. Based on the multivariate Cox regression analysis adjusted for significant potential confounders, RFS was significantly associated with increased mortality risk, with a hazard ratio for death in infants with RFS being 1.74-fold higher compared to those without RFS. Conclusions: Preterm infants born at ≤32 weeks of gestation who develop RFS within the first week of life are at increased risk for both intraventricular hemorrhage and mortality. This study underscores the need for standardized clinical approaches for managing RFS in the neonatal intensive care unit to improve outcomes. Future research should establish a unified RFS definition and conduct clinical trials to optimize parenteral nutrition strategies for this vulnerable population.

## 1. Introduction

Refeeding syndrome (RFS) occurs when nutrition is rapidly reintroduced after a period of prolonged starvation, leading to life-threatening shifts in fluids and electrolytes. This syndrome can cause serious metabolic disturbances, affecting the heart, lungs, blood, and nervous system [1]. The condition was first noted during World War II when individuals recovering from famine unexpectedly fell ill after receiving food. In 1951, Schnitker and colleagues reported that one-fifth of Japanese prisoners who had been starved in prison camps died suddenly after being re-fed and provided with vitamins [2].

During starvation, the body experiences depletion of key nutrients such as potassium and phosphorus [3,4]. When refeeding is started, increased insulin levels drive these electrolytes into the cells, leading to low blood phosphorus and potassium levels [5]. This can cause severe complications such as cardiac arrhythmias, muscle weakness, respiratory failure, convulsions, and encephalopathy. In particular, phosphorus depletion impairs energy production and oxygen delivery to tissues, which can be life-threatening [6,7].

Preterm infants who miss the crucial period of nutrient accumulation in the third trimester of pregnancy are born with insufficient nutrient reserves [8]. Infants who are small-for-gestational-age (SGA) or have intrauterine growth restriction (IUGR) are further at risk of nutrient deficiencies, which are often associated with placental insufficiency [9]. Adequate protein intake immediately after birth is considered essential for supporting growth and neurodevelopment by enhancing protein accretion and activating insulin-like growth factor-I pathways [10,11]. Health providers generally recommend that preterm infants should have a protein intake of 3.5–4.5 g/kg/day [12,13]. However, excessive protein intake can result in metabolic complications including acidosis, hyperammonemia, elevated blood urea nitrogen levels, and RFS [10,14,15]. Our study showed that the incidence rate of RFS in preterm infants born before 32 weeks of gestation is 38% [16]. The reported incidence of RFS in the literature varies widely. For example, in the ProVIDe trial, 20% of extremely low-birth-weight infants presented with RFS. Meanwhile, other studies have reported that the incidence rates of RFS were as high as 90%, particularly in SGA infants, those with IUGR, and those receiving aggressive parenteral nutrition—a high-energy nutrition plan from the first day of life [14,17,18,19,20]. This wide variability in the incidence rates may be attributed to differences in the definition of RFS, population characteristics, and sex. Despite this variability, RFS remains a significant and prevalent condition in preterm infants worldwide.

Several studies have explored the short-term neonatal outcomes associated with RFS, including intraventricular hemorrhage (IVH), bronchopulmonary dysplasia (BPD), and late-onset sepsis (LOS). However, the results are conflicting and inconclusive. Al-Wassia et al. and Cormack et al. found that premature infants who developed RFS had a higher incidence of IVH and severe IVH [14,17]. Conversely, other studies have found no significant association between RFS and IVH [9,19,21,22].

Ross et al. observed that RFS is associated with an increased risk of developing BPD in very-low-birth-weight (VLBW) infants. However, other studies reported no significant difference [9,14,19,21,22,23]. Similarly, Moltu et al. identified an association between severe hypophosphatemia and an increased risk of sepsis in VLBW infants. However, this finding was not supported by other studies, which did not find significant differences in the incidence of LOS [9,14,17,22,23,24,25].

Despite these findings, current studies are limited by several factors. Several studies have small sample sizes, and they included both preterm and term infants and used inconsistent definitions for RFS and hypophosphatemia [20,24,26,27]. In addition, these studies do not often report the concentrations of other parenteral nutrition (PN) components, such as dextrose and proteins [9,17,24]. Due to these limitations, it is challenging to accurately determine the actual clinical outcomes of RFS in preterm infants. Therefore, this study aimed to assess the short-term clinical outcomes of premature infants born at ≤32 weeks of gestation who developed RFS in their first week of life.

## 2. Materials and Methods

### 2.1. Study Design

This study retrospectively performed a review of medical documentation of preterm infants who were admitted to the neonatal intensive care unit (NICU) of King Saud Medical City (KSMC), a tertiary referral center, between January 2015 and June 2024. The average annual number of admissions in the level III NICU at KSMC is 1100.

This study was conducted in accordance with the Declaration of Helsinki and the Good Pharmacoepidemiology Practice Guidelines and was approved by the Medical Ethical Review Committee of KSMC (reference number: H1RI-12-May24-01). The need for informed consent was waived.

### 2.2. Inclusion and Exclusion Criteria

The inclusion criteria were as follows: very-preterm infants with a VLBW (<1500 g) who were born at KSMC, admitted to the level III NICU, and received PN plus lipid emulsion within the first 24 h of life.

The exclusion criteria were as follows: infants with known genetic or chromosomal abnormality, those with congenital infections or significant congenital defects, those who did not receive PN, those who were not born at KSMC or transferred to another hospital or died within the first 7 days after birth, and those who had nonretrievable data.

### 2.3. Data Collection and Follow-Up

The data of infants were collected from birth until death or discharge. The following data were obtained: demographic and clinical characteristics and outcomes, including major morbidities related to prematurity. Further, maternal data, including type of delivery, antenatal steroid treatment, and presence of gestational diabetes mellitus and maternal hypertension, were collected.

### 2.4. Study Outcome

The primary outcome of this study was the short-term clinical outcome of RFS in preterm infants born at ≤32 weeks of gestation.

### 2.5. Definitions

#### Nutrition Protocol

PN: Treatment with PN was started early after birth using starter PN. Individualized PN was prescribed daily. Starter PN contains 10% dextrose, 4% amino acids, and 0.01 mmol/mL of calcium gluconate [1]. Individualized PN solution containing amino acids (3.5–4 g/kg/day), dextrose (5–12 mg/kg/min), Lipid emulsion (1–3 g/kg/day), minerals, sodium chloride (1–3 mmol/kg/day), sodium acetate (1–2 mmol/kg/day), sodium phosphate (1–2 mmol/kg/day), potassium chloride (1–3 mmol/kg/day), potassium acetate (1–2 mmol/kg/day), potassium phosphate (1–2 mmol/kg/day), trace elements (Peditrace^®^), and water- and fat-soluble vitamins (Soluvit^®^ N, and Vitalipid^®^ N Infant; respectively) was started within the first 24 h of life and infused continuously for 24 h [1].

RFS: A clear definition of neonatal RFS has not yet been established. The following definitions were used: hypercalcemia, >2.8 mmol·L^−1^; hypophosphatemia, >1.1 to <1.6 mmol·L^−1^; and severe hypophosphatemia, <1.0 mmol·L^−1^ in the first week of life [14,17,27,28,29].

IVH: IVH was classified into grades I–IV according to the IVH classification of Papile et al. [30]. IVH was diagnosed based on the findings of head ultrasound performed between days 5 and 7 after birth [31,32]. All ultrasonography scans were performed by one expert radiologist and checked by another expert radiologist. 7.5- and 10-MHz transducers (LOGIQ e; GE Medical Systems Co., Ltd., Nanjing, China) were used to perform ultrasonography in sagittal and coronal planes.

### 2.6. Statistical Analysis

Before the analysis, the dataset was reviewed and checked for missing data. Data were analyzed using the Statistical Package for the Social Sciences software for Windows version 25.0 (IBM Corp., Armonk, NY, USA).

Infant and maternal variables were presented as descriptive statistics (median, interquartile range, frequency, and percentage). The Mann–Whitney U test was used for between-group comparisons of ordinal qualitative variables. The Fisher’s exact test was utilized to determine the association between categorical variables. The unpaired Student’s *t*-test was used for between-group comparisons of continuous variables with a normal distribution. The Mann–Whitney U test was used to assess variables with a non-normal distribution. The Kolmogorov–Smirnov test and a visual inspection of histograms were performed to evaluate the distribution of quantitative variables.

To determine the association between RFS in premature infants and neonatal outcomes, a univariate relative risk analysis of the recorded variables (gestational age, birth weight, SGA, delivery mode, sex, 1- and 5-min Apgar scores, maternal hypertension, antenatal steroid treatment, premature rupture of the membrane, gestational diabetes mellitus, necrotizing enterocolitis, surfactant use, LOS, dextrose intake, amino acids, and lipid emulsion, phosphate intake) was initially performed because the abovementioned factors were considered potential confounders. All factors with a *p*-value of <0.05 in the univariate analysis were included in the final multivariate regression analysis. The modified log Poisson regression with generalized linear models and a robust variance estimator (Huber–White) were applied in the univariate relative risk analysis and to the models to adjust the relative risk for the association between RFS and neonatal outcomes. All statistical tests were two-tailed, and *p*-values of <0.05 were considered significant.

## 3. Results

**Figure 1:**
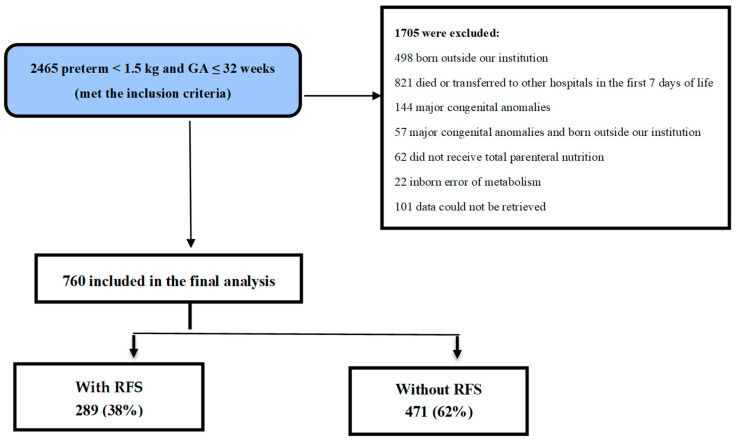
Flow chart of patient selection. *GA* gestational age.

In total, 2465 preterm infants with a gestational age of ≤32 weeks and a birth weight of <1500 g were admitted to the level 3 NICU. Of them, 760 met the inclusion criteria and were eligible for the final analysis (Figure 1).

Further, 289 (38%) of 760 infants developed RFS. Of 760 patients, 264 were aged <28 gestational weeks. Table 1 and Table 2 show the demographic characteristics of the mothers and infants stratified according to RFS.

Infants with RFS had a lower gestational age and birth weight, shorter length, and smaller head circumference than those without RFS (*p* < 0.001).

**Figure 2:**
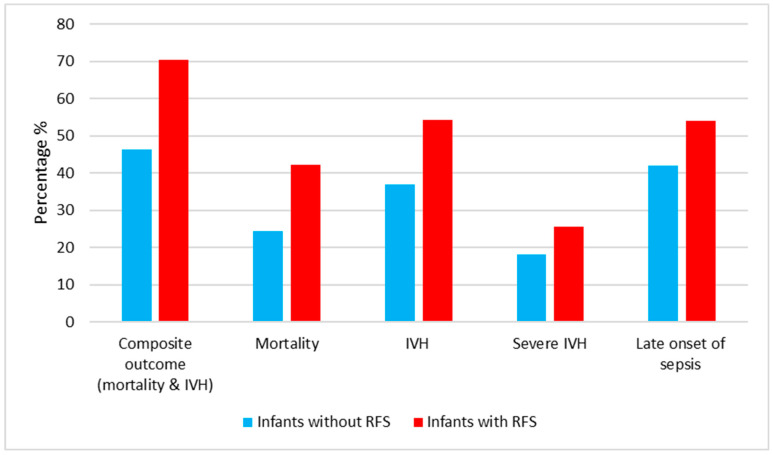
Percentage of neonatal morbidities and mortality stratified according to RFS.

Infants with RFS had lower Apgar scores at 1 and 5 min than infants without RFS (*p* < 0.001). Moreover, male infants were at higher risk of RFS than female infants (*p* < 0.001). In addition, infants who developed RFS required more surfactant than those without RFS (*p* < 0.001). Moreover, there was a significant association between average lipid intake, amino acid intake, phosphate intake, TPN duration, and RFS. Infants with RFS received more lipid, more amino acid, less phosphate, and more TPN than those without RFS (*p* = 0.02, 0.01, 0.01, and 0.002; respectively). Figure 2 shows the percentage of infants with RFS and neonatal outcomes.

In the univariate analysis, RFS was significantly associated with neonatal morbidity and mortality in very-preterm infants (Table 3). In infants aged < 32 gestational weeks, RFS was significantly associated with composite outcome, mortality, IVH, severe IVH, LOS, and BPD (*p* < 0.001, <0.001, <0.001, <0.001, <0.001, and 0.02, respectively) (Table 3). There was no significant difference between RFS and infants’ growth anthropometrics. Moreover, no association was observed between RFS and the length of invasive ventilator as well as the length of hospital stay (*p* = 0.33, 0.05; respectively).

Based on the multivariate regression analysis performed after adjusting the variables that were significant in the univariate analysis, RFS was significantly associated with composite outcome and IVH (adjusted relative risk [aRR]: 1.70, 95% confidence interval [CI]: 1.45–2.0; aRR: 1.59, 95% CI: 1.31–1.94, respectively) (Table 4).

**Figure 3:**
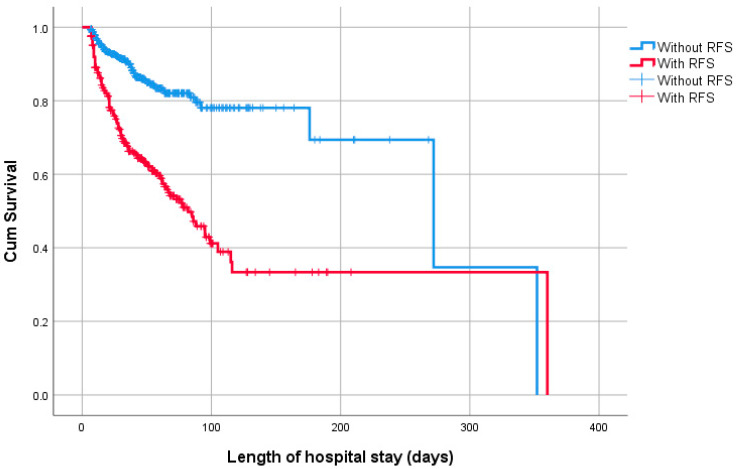
Kaplan–Meier curves showing the association between RFS and survival among infants aged < 32 gestational weeks, log-rank test, *p* < 0.001.

Figure 3 shows the Kaplan–Meier curves of the association between RFS and survival among all infants (log-rank test, *p* < 0.001). The multivariate Cox regression curve adjusted for significant potential confounders showed a significant association between RFS and survival. Moreover, the hazard ratios (HRs) for death in infants with RFS increased by 1.74-fold (95% CI: 1.37–2.21) than those in infants without RFS (Table 4).

In the subanalysis, RFS was significantly associated with composite outcome, mortality, IVH, and NEC (medical) in newborns with gestational age < 28 gestational weeks (*p* < 0.001, *p* < 0.001, *p* = 0.001, and *p* = 0.02, respectively) (Table 3). The adjusted model of the multivariate regression analysis showed a significant association between RFS as well as composite outcome and IVH (aRR: 1.40, 95% confidence interval [CI]: 1.17–1.68); aRR: 1.30, 95% CI: 1.06–1.65, respectively) (Table 4).

**Figure 4:**
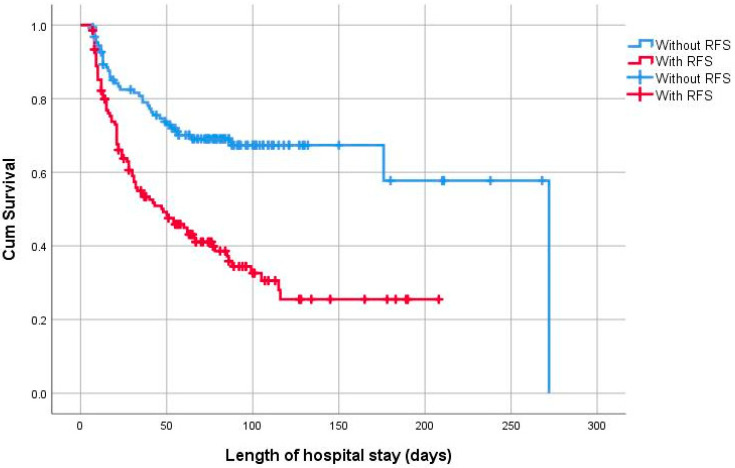
Kaplan–Meier curves showing the association between RFS and survival among infants aged < 28 gestational weeks, log-rank test, *p* < 0.001.

Figure 4 shows the Kaplan–Meier curves of the association between RFS and survival among infants aged < 28 gestational weeks (log-rank test, *p* < 0.001). In newborns with gestational age < 28 gestational weeks, the multivariable Cox regression curve adjusted for significant potential confounders showed that the HRs for death in infants with RFS increased by 1.59-fold (95% CI: 1.20–2.09) compared with those in infants without RFS (Table 4).

## 4. Discussion

The current study showed that preterm infants born at ≤32 weeks of gestation who developed RFS within the first week of life had a higher incidence of IVH and/or mortality during their hospital stay. However, there was no significant difference in the duration of mechanical ventilation, the rate of BPD, and the incidence of LOS between infants who developed RFS and those who did not.

Our findings are in accordance with those of the ProVIDe trial, which revealed that the incidence of mortality increased by 3-fold in infants with RFS and that severe hypophosphatemia was associated with a 5-fold increase in the rate of IVH [14]. In our study, the mortality rate of infants with RFS was approximately 42%. Meanwhile, despite using the same definition of RFS, the mortality rate in the ProVIDe trial was 32%. In addition, the mortality rate in the non-RFS population was similar between the two studies, with 13% in the current cohort versus 11% in the ProVIDe trial. In our previous study investigating the incidence and risk factors of RFS in preterm infants, IVH was found to be a significant risk factor for the development of RFS [16]. Considering that the majority of IVH cases occur within the first week of life, the temporal sequence between the onset of IVH and RFS is challenging to determine. Nonetheless, our findings showed a strong association between these two conditions in preterm infants. These results are in accordance with those of the ProVIDe trial, and the study conducted by Al-Wassia et al. also revealed a significant association between IVH and the development of RFS [14,17].

In contrast, some studies did not observe a significant difference in the incidence of mortality and IVH associated with RFS. These studies often had limitations. For example, they had small sample sizes, a low incidence of IVH, different definitions of RFS, and variations in the composition of TPN, which might have influenced their findings [9,19,21,22,24]. We did not find a significant difference in the rate of LOS between the two groups, a result consistent with the findings of other studies, including the ProVIDe trial [9,14,17,22,23,25]. However, Moltu et al. reported that VLBW infants with RFS had a high rate of LOS [24].

There was no convincing evidence showing that RFS is associated with a longer duration of mechanical ventilation or a higher rate of BPD. Our findings on the length of mechanical ventilation and BPD are in accordance with those of Ross et al. and Igarashi et al. [9,21] as well as the ProVIDe trial and study of Bustos et al., respectively [14,23]. However, other studies, such as those by Sung et al. and Ross et al., have reported associations between RFS and the duration of mechanical ventilation, and the incidence rate of BPD, respectively [9,22].

The discrepancies in the results of published papers could be attributed to different risk factors present in each population. In our study, male sex, IVH, and low sodium phosphate intake within the first week were significant risk factors for developing RFS in preterm infants [16]. Other studies have revealed that IUGR and SGA status are factors associated with an increased risk and severity of RFS [9,20,26,27]. In addition, some studies have found that the incidence of RFS has increased after the implementation of recommendations to increase amino acid intake within the first days of life [14,24,33].

Although there are differences among various studies, including our own, it is more important to recognize the risks associated with RFS in preterm infants and its potential short- and long-term complications. To address RFS effectively, it is important to establish a unified RFS definition in the NICU, which could help eliminate variations worldwide. In addition, clinical trials should be performed to determine the optimal balance of PN. Meanwhile, clinicians should implement a standardized approach in the NICU, involving a multidisciplinary team to facilitate the following: (1) identify at-risk infants; (2) develop individualized PN strategies; (3) establish guidelines for early enteral nutrition; and (4) create a laboratory monitoring protocol. These steps are essential, considering our findings, which show a significant association between RFS and adverse outcomes such as IVH and an increased rate of mortality.

Our current study was based on previous research on the incidence and risk factors of RFS in preterm infants, with a focus on highlighting the short-term complications associated with RFS in this population. This was a retrospective study conducted in a single tertiary center. However, it included a substantial number of patients who are at risk of developing RFS. In addition, infants with IUGR in this study were not identified, as this requires mothers to be closely followed throughout early antenatal care.

## 5. Conclusions

Preterm infants born at ≤32 weeks of gestation who develop RFS within the first week of life are at higher risk of IVH, mortality, and longer duration of TPN. These findings emphasize the urgent need for the early identification and standardized management of RFS in the NICU.

## Figures and Tables

**Table 1:** Maternal characteristics of the participants stratified according to RFS.

Gestational Age	<32 Weeks				<28 Weeks			
Parameters	N	Infants without Refeeding Syndrome (n = 471)	Infants with Refeeding Syndrome (n = 289)	*p*-Value	N	Infants without Refeeding Syndrome (n = 128)	Infants with Refeeding Syndrome (n = 136)	*p*-Value
Antenatal steroid treatment, n (%)	760	253 (53.7)	147 (50.9)	0.45	264	70 (54.7)	71 (52.2)	0.71
Gestational diabetes mellitus, n (%)	760	27 (4.4)	14 (5.9)	0.37	264	7 (5.5)	4 (2.9)	0.36
Maternal hypertension, n (%)	760	119 (25.3)	67 (23.2)	0.54	264	26 (20.3)	27 (19.9)	1
Preterm rupture of membrane, n (%)	760	59 (13.1)	20 (7.2)	0.01 *	264	17 (13.3)	12 (8.8)	0.33
Cesarean section, n (%)	760	223 (47.3)	146 (50.5)	0.41	264	72 (56.3)	75 (55.1)	0.90

* *p*-values < 0.05.

**Table 2:** Neonatal characteristics of the participants stratified according to RFS.

Gestational Age	<32 Weeks				<28 Weeks			
Parameters	N	Infants without Refeeding Syndrome (n = 471)	Infants with Refeeding Syndrome (n = 289)	*p*-Value	N	Infants without Refeeding Syndrome (n = 128)	Infants with Refeeding Syndrome (n = 136)	*p*-Value
Gestational age (weeks), median (IQR)	760	29 (27.0–31.0)	28 (26–30.0)	<0.001 *	264	26 (25.0–27.0)	26 (25.0–27.0)	0.02 *
Birth weight (grams), (IQR)	760	1180 (950–1370)	950 (765–1200)	<0.001 *	264	845 (711.25–960)	775 (661.25–905)	0.008 *
Length (cm), median (IQR)	760	38 (35–40)	35 (32–38)	<0.001 *	264	33 (31–35)	33 (31–35)	0.10
Head circumference (cm), (IQR)	760	27 (25–28)	25 (23–27)	<0.001 *	264	24 (23–25)	23 (22–25)	0.03 *
1-min Apgar score, median (IQR)	760	6 (4–7)	5 (3–6)	<0.001 *	264	5 (3–6)	4 (2–6)	0.03 *
5-min Apgar score, median (IQR)	760	7 (6–8)	7 (7–8)	<0.001 *	264	5 (6–7)	4 (6–7)	0.17
Male sex, n (%)	760	221 (46.9)	174 (60.2)	<0.001 *	264	70 (54.7)	90 (66.2)	0.06
Expressed breast milk	760	247 (52.4)	145 (50.2)	0.55	264	75 (58.6)	64 (47.1)	0.06
Noninvasive respiratory support, n (%)	760	416 (88.3)	218 (75.4)	<0.001 *	264	96 (75)	79 (58.1)	0.004 *
Respiratory distress syndrome requiring surfactant, n (%)	760	281 (59.7)	224 (77.5)	<0.001 *	264	120 (93.8)	128 (94.1)	1
Patent ductus arteriosus requiring treatment, n (%)	760	35 (7.4)	34 (11.8)	0.05	264	24 (18.8)	28 (20.6)	0.76
Peripherally inserted central catheter (PICC), n (%)	760	154 (32.7)	121 (41.9)	0.01 *	264	60 (46.9)	75 (55.1)	0.22
Umbilical arterial catheter (UAC), n (%)	760	134 (28.5)	117 (40.5)	0.001 *	264	71 (55.5)	73 (53.7)	0.81
Umbilical venous catheter (UVC), n (%)	760	356 (75.6)	250 (86.5)	<0.001 *	264	121 (94.5)	125 (91.9)	0.47
Central venous catheter (CVC), n (%)	760	22 (4.7)	25 (8.7)	0.03 *	264	17 (13.3)	17 (12.5)	0.86
Average parenteral lipid intake within the first 7 days (g/kg/day), median (IQR)	760	2 (1.54–2.35)	2.1 (1.67–2.5)	0.02 *	264	1.8 (1.4–2.3)	1.8 (1.3–2.4)	0.95
Average parenteral protein intake within the first 7 days (g/kg/day), median (IQR)	760	3.90 (3.67–4.0)	3.97 (3.75–4.0)	0.01 *	264	4 (3.7–4.0)	4 (3.8–4.0)	0.13
Average parenteral carbohydrate intake within the first 7 days (mg/kg/min), median (IQR)	760	8.47 (7.74–9.12)	8.46 (7.65–9.04)	0.55	264	8 (7–8.8)	7.7 (6.4–8.5)	0.03 *
Average parenteral phosphate intake within the first 7 days (mg/kg/min), median (IQR)	760	0.30 (0–0.6)	0.24 (0–0.4)	0.01 *	264	0.30 (0.02–0.62)	0.2 (0–0.44)	0.03 *
TPN duration, median (IQR)	760	14 (7–29)	18 (9–35)	0.002 *	264	28 (12–48)	23 (11–41)	0.45

* *p*-values < 0.05.

**Table 3:** Univariate analysis of growth anthropometrics, neonatal morbidity, and mortality stratified according to RFS.

Variables	<32 Weeks				<28 Weeks			
	N	Infants without Refeeding Syndrome (n = 471)	Infants with Refeeding Syndrome (n = 289)	*p*-Value	N	Infants without Refeeding Syndrome (n = 128)	Infants with Refeeding Syndrome (n = 136)	*p*-Value
Growth anthropometrics								
Weight gain velocity (g/kg/day), median (IQR)	574	6.7 (5.23–8.26)	6.78 (4.89–8.09)	0.50	141	6.16 (5.33–7.59)	6.61 (4.99–8.04)	0.72
Weight at discharge (g), median (IQR)	574	1850 (1770–2026)	1870 (1750–2020)	0.94	141	1935 (1790–2191)	1920 (1735–2347)	0.87
Z-score for weight at discharge, median (IQR)	574	−2.02 (−2.86 to −1.26)	−1.98 (−3.20 to −1.35)	0.41	141	−2.46 (−3.47 to −1.31)	−2.0 (−3.32 to −1.29)	0.40
Neonatal morbidities and mortality								
Composite outcome (IVH, mortality)	760	148 (31.4)	204 (70.5)	<0.001 *	264	73 (57)	120 (88.2)	<0.001 *
Mortality, n (%)	760	64 (13.6)	122 (42.2)	<0.001 *	264	39 (30.5)	84 (61.8)	<0.001 *
Intraventricular hemorrhage, n (%)	760	124 (26.3)	157 (54.3)	<0.001*	264	64 (50)	96 (70.6)	0.001 *
Severe intraventricular hemorrhage, n (%)	760	63 (13.3)	74 (25.6)	<0.001 *	264	41 (32)	56 (41.2)	0.15
Early-onset sepsis (culture-proven), n (%)	760	6 (1.3)	5 (1.7)	0.35	264	2 (1.6)	4 (2.9)	0.68
Late-onset sepsis (culture-proven), n (%)	760	164 (34.8)	156 (54)	<0.001 *	264	78 (60.9)	97 (71.3)	0.09
Necrotizing enterocolitis (stage ≥ 2), n (%):								
Medical management	760	147 (31.2)	95 (32.8)	0.63	264	61 (47.7)	46 (33.8)	0.02 *
Surgical management	760	30 (6.4)	19 (6.6)	1	264	18 (14.1)	15 (11)	0.46
Spontaneous intestinal perforation, n (%)	760	5 (1.1)	5 (1.7)	0.52	264	3 (2.3)	3 (2.2)	1
Bronchopulmonary dysplasia, n (%)	511	132 (40.4)	94 (51.1)	0.02 *	176	70 (72.9)	60 (75)	0.86
Duration of invasive ventilation, days, median (IQR)	760	11 (2–27)	13 (6–31)	0.3	264	13 (3–31)	17 (8–34)	0.29
Length of hospital stay after excluding death infants, median (IQR)	574	43 (26–67)	49 (31–73)	0.05	141	82 (62–102)	75 (55–105)	0.61

* *p*-values < 0.05.

**Table 4:** Multivariate regression for infant’s outcome stratified according to RFS.

	ALL Neonates		Newborns with Gestational Age < 28 Weeks	
Variables	Unadjusted 95% CI	Adjusted 95% CI	Unadjusted 95% CI	Adjusted 95% CI
Composite outcome (IVH, mortality) (RR)	2.25 (1.94–2.63)	1.70 (1.45–2.0)	1.55 (1.32–1.82)	1.40 (1.17–1.68)
Mortality (HR)	3.21 (2.37–4.34)	1.74 (1.37–2.21)	2.57 (1.75–3.77)	1.59 (1.20–2.09)
Intraventricular hemorrhage (RR)	2.07 (−9.08 to 13.22)	1.59 (1.31–1.94)	1.41 (1.15–1.73)	1.30 (1.06–1.65)
Severe intraventricular hemorrhage (RR)	1.93 (1.43–2.61)	1.24 (0.90–1.70)	1.28 (0.93–1.76)	----------
Bronchopulmonary dysplasia (RR)	1.27 (1.04–1.54)	0.86 (0.72–1.03)	1.03 (0.86–1.23)	----------
Late-onset sepsis (RR)	1.55 (1.32–1.83)	1.16 (0.98–1.39)	1.17 (0.98–1.3)	----------

## Data Availability

The original contributions presented in the study are included in the article, further inquiries can be directed to the corresponding author.

## References

[1] da Silva J.S.V., Seres D.S., Sabino K., Adams S.C., Berdahl G.J., Citty S.W., Cober M.P., Evans D.C., Greaves J.R., Gura K.M. (2020). Parenteral Nutrition Safety and Clinical Practice Committees, American Society for Parenteral and Enteral Nutrition. ASPEN consensus recommendations for refeeding syndrome. Nutr. Clin. Pract..

[2] Schnitker M.A., Mattman P.E., Bliss T.L. (1951). A clinical study of malnutrition in Japanese prisoners of war. Ann. Intern. Med..

[3] Finn P.F., Dice J.F. (2006). Proteolytic and lipolytic responses to starvation. Nutrition.

[4] Cahill G.F. (2006). Fuel metabolism in starvation. Annu. Rev. Nutr..

[5] Friedli N., Stanga Z., Sobotka L., Culkin A., Kondrup J., Laviano A., Mueller B., Schuetz P. (2017). Revisiting the refeeding syndrome: Results of a systematic review. Nutrition.

[6] Goretti Penido M., Alon U.S. (2012). Phosphate homeostasis and its role in bone health. Pediatr. Nephrol..

[7] Sharma S., Brugnara C., Betensky R.A., Waikar S.S. (2015). Reductions in red blood cell 2,3-diphosphoglycerate concentration during continuous renal replacment therapy. Clin. J. Am. Soc. Nephrol..

[8] Kovacs C.S. (2016). Maternal mineral and bone metabolism during pregnancy, lactation, and post-weaning recovery. Physiol. Rev..

[9] Ross J.R., Finch C., Ebeling M., Taylor S.N. (2013). Refeeding syndrome in very-low-birth-weight intrauterine growth-restricted neonates. J. Perinatol..

[10] Amissah E.A., Brown J., Harding J.E. (2018). Protein supplementation of human milk for promoting growth in preterm infants. Cochrane Database Syst. Rev..

[11] Bellagamba M.P., Carmenati E., D’Ascenzo R., Malatesta M., Spagnoli C., Biagetti C., Burattini I., Carnielli V.P. (2016). One extra gram of protein to preterm infants from birth to 1800 g: A single-blinded randomized clinical trial. J. Pediatr. Gastroenterol. Nutr..

[12] Agostoni C., Buonocore G., Carnielli V.P., De Curtis M., Darmaun D., Decsi T., Domellöf M., Embleton N.D., Fusch C., Genzel-Boroviczeny O. (2010). Enteral nutrient supply for preterm infants: Commentary from the European Society of Paediatric Gastroenterology, Hepatology and Nutrition Committee on Nutrition. J. Pediatr. Gastroenterol. Nutr..

[13] Embleton N.D., Jennifer Moltu S., Lapillonne A., van den Akker C.H.P., Carnielli V., Fusch C., Gerasimidis K., van Goudoever J.B., Haiden N., Iacobelli S. (2023). Enteral nutrition in preterm infants (2022): A position paper from the ESPGHAN Committee on Nutrition and invited experts. J. Pediatr. Gastroenterol. Nutr..

[14] Cormack B.E., Jiang Y., Harding J.E., Crowther C.A., Bloomfield F.H., ProVIDe Trial Group (2021). Neonatal refeeding syndrome and clinical outcome in extremely low-birth-weight babies: Secondary cohort analysis from the ProVIDe trial. JPEN J. Parenter. Enter. Nutr..

[15] Weintraub A.S., Blanco V., Barnes M., Green R.S. (2015). Impact of renal function and protein intake on blood urea nitrogen in preterm infants in the first 3 weeks of life. J. Perinatol..

[16] Asfour S.S., Alshaikh B., Mathew M., Fouda D.I., Al-Mouqdad M.M. (2024). Incidence and risk factors of refeeding syndrome in preterm infants. Nutrients.

[17] Al-Wassia H., Lyon A.W., Rose S.M., Sauve R.S., Fenton T.R. (2019). Hypophosphatemia is prevalent among preterm infants less than 1,500 grams. Am. J. Perinatol..

[18] Brener Dik P.H., Galletti M.F., Bacigalupo L.T., Fernández Jonusas S., Mariani G.L. (2018). Hypercalcemia and hypophosphatemia among preterm infants receiving aggressive parenteral nutrition. Arch. Argent. Pediatr..

[19] Pająk A., Królak-Olejnik B., Szafrańska A. (2018). Early hypophosphatemia in very low birth weight preterm infants. Adv. Clin. Exp. Med..

[20] Chaudhary V., Jayendra R.G., Shreya A.P. (2020). Hypophosphatemia in refeeding syndrome in intrauterine growth restricted IUGR neonates who are receiving nutrition: A prospective observational study. Asian J. Pediatr. Res..

[21] Igarashi A., Okuno T., Ohta G., Tokuriki S., Ohshima Y. (2017). Risk factors for the development of refeeding syndrome-like hypophosphatemia in very low birth weight infants. Dis. Markers.

[22] Sung S.I., Chang Y.S., Choi J.H., Ho Y., Kim J., Ahn S.Y., Park W.S. (2019). Increased risk of refeeding syndrome-like hypophosphatemia with high initial amino acid intake in small-for-gestational-age, extremely-low-birthweight infants. PLoS ONE.

[23] Bustos Lozano G., Soriano-Ramos M., Pinilla Martín M.T., Chumillas Calzada S., García Soria C.E., Pallás-Alonso C.R. (2019). Early hypophosphatemia in high-risk preterm infants: Efficacy and safety of sodium glycerophosphate from first day on parenteral nutrition. JPEN J. Parenter. Enteral Nutr..

[24] Moltu S.J., Strømmen K., Blakstad E.W., Almaas A.N., Westerberg A.C., Brække K., Rønnestad A., Nakstad B., Berg J.P., Veierød M.B. (2013). Enhanced feeding in very-low-birth-weight infants may cause electrolyte disturbances and septicemia—A randomized, controlled trial. Clin. Nutr..

[25] Brener Dik P.H., Galletti M.F., Fernández Jonusas S.A., Alonso G., Mariani G.L., Fustiñana C.A. (2015). Early hypophosphatemia in preterm infants receiving aggressive parenteral nutrition. J. Perinatol..

[26] Boubred F., Herlenius E., Bartocci M., Jonsson B., Vanpée M. (2015). Extremely preterm infants who are small for gestational age have a high risk of early hypophosphatemia and hypokalemia. Acta Paediatr..

[27] Ichikawa G., Watabe Y., Suzumura H., Sairenchi T., Muto T., Arisaka O. (2012). Hypophosphatemia in small for gestational age extremely low birth weight infants receiving parenteral nutrition in the first week after birth. J. Pediatr. Endocrinol. Metab..

[28] Senterre T., Abu Zahirah I., Pieltain C., de Halleux V., Rigo J. (2015). Electrolyte and mineral homeostasis after optimizing early macronutrient intakes in VLBW infants on parenteral nutrition. J. Pediatr. Gastroenterol. Nutr..

[29] Mbethe A.P., Mda S. (2017). Incidence of refeeding syndrome and its associated factors in South African children hospitalized with severe acute malnutrition. Iran. J. Pediatr..

[30] Papile L.A., Burstein J., Burstein R., Koffler H. (1978). Incidence and evolution of subependymal and intraventricular hemorrhage: A study of infants with birth weights less than 1,500 gm. J. Pediatr..

[31] Ballabh P. (2010). Intraventricular hemorrhage in premature infants: Mechanism of disease. Pediatr. Res..

[32] Huvanandana J., Nguyen C., Thamrin C., Tracy M., Hinder M., McEwan A.L. (2017). Prediction of intraventricular haemorrhage in preterm infants using time series analysis of blood pressure and respiratory signals. Sci. Rep..

[33] Bonsante F., Iacobelli S., Latorre G., Rigo J., De Felice C., Robillard P.Y., Gouyon J.B. (2013). Initial amino acid intake influences phosphorus and calcium homeostasis in preterm infants—It is time to change the composition of the early parenteral nutrition. PLoS ONE.

